# Evaluation of Factors Affecting Fluoride Release from Fluoride Varnishes: A Systematic Review

**DOI:** 10.3390/ma18194603

**Published:** 2025-10-04

**Authors:** Maciej Dobrzyński, Agnieszka Kotela, Sylwia Klimas, Zuzanna Majchrzak, Julia Kensy, Marzena Laszczyńska, Mateusz Michalak, Zbigniew Rybak, Magdalena Fast, Jacek Matys

**Affiliations:** 1 Department of Pediatric Dentistry and Preclinical Dentistry, Wroclaw Medical University, Krakowska 26, 50-425 Wroclaw, Poland; sylwia.klimas@student.umw.edu.pl; 2 Medical Center of Innovation, Wroclaw Medical University, Krakowska 26, 50-425 Wroclaw, Poland; kotela.agnieszka@gmail.com (A.K.); marzenalaszczynska@gmail.com (M.L.); mateusz.michalak92@gmail.com (M.M.); 3 Faculty of Dentistry, Wroclaw Medical University, Krakowska 26, 50-425 Wroclaw, Poland; julia.kensy@student.umw.edu.pl; 4 Pre-Clinical Research Centre, Wroclaw Medical University, Bujwida 44, 50-345 Wroclaw, Poland; zbigniew.rybak@umw.edu.pl; 5 Department of Drug Form Technology, Wroclaw Medical University, Borowska 211 A, 50-556 Wroclaw, Poland; magdalena.fast@umw.edu.pl; 6 Dental Surgery Department, Wroclaw Medical University, Krakowska 26, 50-425 Wroclaw, Poland

**Keywords:** additives, dentistry, fluoride release, prevention, varnish

## Abstract

Introduction: Fluoride varnishes are widely used in caries prevention, but the rate and duration of fluoride ion release differ depending on material composition and environmental factors. Objectives: This systematic review synthesized evidence from in vitro studies on human teeth to identify key factors influencing fluoride release. Methods: A systematic literature search was conducted in July 2025 in PubMed, Scopus, Web of Science, Embase, and the Cochrane Library using the terms “fluoride release” AND “varnish” in titles and abstracts. Study selection followed PRISMA 2020 guidelines, predefined eligibility criteria, and was structured according to the PICO framework. Of 484 retrieved records, 15 studies met the inclusion criteria and were analyzed qualitatively. Results: The primary outcome was the magnitude and duration of fluoride release from varnishes. Most studies reported peak release within the first 24 h, followed by a marked decline, although some formulations (e.g., Clinpro XT and Duraphat) maintained more stable long-term release. Substantial methodological heterogeneity was observed across studies, including differences in sample type, storage medium, pH, temperature, and measurement protocols, which influenced fluoride release dynamics. Reported secondary outcomes included enamel remineralization, changes in surface properties, and antibacterial activity, with bioactive additives such as CPP–ACP and TCP enhancing preventive effects. Acidic conditions consistently increased fluoride release. Conclusions: The magnitude and persistence of fluoride release from varnishes depend on both intrinsic material properties and external environmental conditions. Bioactive additives may prolong fluoride availability and provide additional preventive benefits.

## 1. Introduction

Fluoride plays a crucial role in the prevention of dental caries [1]. Its anticariogenic effect is achieved through several mechanisms, including the enhancement of enamel resistance by promoting remineralization [2], the inhibition of demineralization, and the suppression of cariogenic bacteria [3,4,5]. Fluoride ions are incorporated into hydroxyapatite crystals, forming fluorapatite, which exhibits greater resistance to acid dissolution in the oral environment [6,7,8]. In addition, fluoride interferes with bacterial enzymatic activity, thereby reducing acid production within the dental biofilm [2,9]. It also plays an important role in the process of amelogenesis [10,11]. Clinical studies, including randomized controlled trials, have demonstrated caries reduction ranging from 15% to over 60%, depending on the fluoride concentration, frequency of application, and patient risk profile [9,12,13]. The wide availability of fluoride formulations—such as toothpastes and mouth rinses—ensures convenient daily access for patients [14,15,16]. High-risk groups, including individuals with xerostomia, those undergoing radiotherapy, or patients with orthodontic appliances, particularly benefit from higher-concentration fluoride products to minimize treatment-related side effects [17,18,19,20]. From a public health perspective, fluoride remains one of the most cost-effective and broadly recommended agents for caries prevention worldwide [14,15,21].

Fluoride varnishes are among the most widely used fluoride-based formulations because of their multiple benefits and ease of application. They are preferred by clinicians for the prevention of both primary and secondary caries [1,22,23], the remineralization of early carious lesions [2], and the restoration of enamel integrity after orthodontic treatment in both children and adults [24,25,26,27,28]. When applied, the varnish hardens upon contact with saliva, forming a protective layer on the tooth surface that gradually releases fluoride ions [3,29,30]. This continuous, slow release provides greater benefits than single high-dose applications, such as those from gels or foams [31]. In addition, varnishes may be supplemented with agents such as xylitol or calcium phosphates, which enhance their anticariogenic properties [3,22,32]. When used in appropriate amounts (0.1 mL in children up to 1 year of age, 0.25 mL during the primary dentition stage, 0.4 mL during mixed dentition, and 0.75 mL in adults) and at suitable intervals (every 3–6 months, depending on the clinical condition), fluoride varnish is a safe and effective method of caries prevention [25,33]. Contraindications include hypersensitivity to any component, bronchial asthma, and stomatitis [9,34,35].

**Figure 1:**
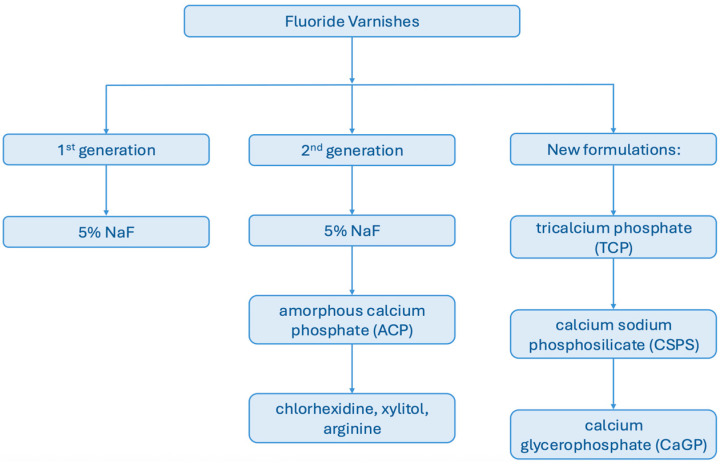
Generations of Fluoride Varnishes.

Fluoride varnishes can be classified into generations according to the progressive refinement of their formulations. The first generation is represented by preparations containing 5% sodium fluoride (NaF), corresponding to 2.26% (22,600 ppm) fluoride, which has long been considered the gold standard in caries prevention [9,36]. These varnishes act primarily through sustained fluoride release and the formation of fluorapatite, thereby increasing enamel resistance to acid attacks [27]. The second generation of varnishes builds upon this base by incorporating amorphous calcium phosphate (ACP), which provides an additional source of calcium and phosphate ions [37,38]. This combination not only enhances the remineralization process but also helps to inhibit enamel demineralization in acidic conditions [1,2,39]. Some products are further supplemented with bioactive compounds such as chlorhexidine, xylitol, or arginine, which contribute antimicrobial, anticariogenic, or biofilm-modulating effects [40,41,42]. The most recent generation of varnishes includes advanced formulations with functional additives such as tricalcium phosphate (TCP), calcium sodium phosphosilicate (CSPS, also known as bioactive glass), and calcium glycerophosphate (CaGP) [9]. These compounds are designed to optimize ion release profiles, provide longer-term remineralization, and in some cases stimulate salivary buffering capacity. However, despite their promising properties, many of these novel products remain under clinical investigation, and long-term evidence regarding their efficacy and safety is still limited (see Figure 1).

It is also worth noting that the release of fluoride from dental products is influenced by several factors. For example, calcium ions present in saliva can bind to fluoride ions, reducing their availability. In turn, acidic pH increases salt solubility, leading to a higher fluoride concentration in solution [43]. The type of matrix in which fluoride occurs is also important. Studies confirm that amine fluorides are highly stable and, unlike free ions derived from sources such as NaF, do not react quickly with cations in saliva. This makes fluoride more effective [44]. Functional additives significantly impact the kinetics and release of fluoride from varnishes. The presence of CPP-ACP or fTCP in varnishes increases fluoride release time, thereby enhancing the effectiveness of the additive. Another effective but lesser-known additive is CXP, which increases fluoride release by up to four times when added to varnish [45,46]

A review of the available literature on factors affecting fluoride release from varnishes revealed a lack of systematic analyses in this field. The included studies showed considerable heterogeneity, particularly regarding the composition of the storage medium, the concentration of the tested varnishes, the application protocols, and the timing of fluoride release measurements. Although individual studies examined selected factors influencing fluoride release, the overall evidence underscores the need to organize and synthesize this information. The aim of this study was to evaluate the factors that influence fluoride release from fluoride varnishes. This systematic review provides a structured analysis of existing research and delivers clinically relevant, evidence-based information to enhance the effectiveness of varnish-based preventive and therapeutic strategies. A unique feature of this review is its exclusive focus on studies conducted on human teeth, which ensures that the findings are as closely aligned with real clinical conditions as possible, while at the same time encompassing both the evaluation of fluoride release and the assessment of the remineralization potential of the varnishes.

## 2. Materials and Methods

### 2.1. Focused Question

In fluoride varnishes used in dentistry (P), how do variations in formulation (e.g., fluoride concentration or additives such as CPP–ACP, ACP, TCP, or DCPD) (I), compared with first-generation fluoride varnishes or untreated controls (C), influence the magnitude and pattern of fluoride release (O)?

### 2.2. Information Sources, Search Strategy and Study Design

In July 2025, a wide-ranging literature search was performed in five electronic databases: PubMed, Scopus, Web of Science (WoS), Embase, and the Cochrane Library, to identify studies meeting the established inclusion criteria. The search strategy was designed to capture research specifically investigating factors influencing fluoride release from fluoride varnishes. Therefore, it was restricted to titles and abstracts containing both the terms “fluoride release” and “varnish.” No restrictions regarding publication year were applied. Only studies with available full-text versions were considered for inclusion.

The exact search strategies used in each database were as follows:

PubMed: (“fluoride release”[Title/Abstract]) AND (“varnish”[Title/Abstract]);
Scopus: TITLE-ABS-KEY (“fluoride release” AND varnish);
Web of Science (WoS): TS = (“fluoride release” AND varnish);
Embase: (‘fluoride release’:ab,ti) AND (varnish:ab,ti);
Cochrane Library: (“fluoride release” in Title, Abstract, Keywords) AND varnish.

### 2.3. Eligibility Criteria

The researchers chose to include only those articles that met the following criteria [47,48,49,50,51,52]:

- Investigation of evaluation the fluoride release from dental varnishes;
- Only research articles;
- In vitro studies;
- Studies conducted only on human teeth;
- Studies in English;
- Full-text articles.

The exclusion criteria the reviewers agreed upon were as follows [47,48,49,50,51,52]:

- Evaluation of properties other than fluoride release;
- In vivo studies;
- Studies conducted on animal teeth or synthetic samples;
- Clinical reports;
- Review articles;
- Editorial papers;
- Full text not accessible;
- Duplicated publications.

The year of publication was not restricted.

### 2.4. Data Collection Process and Data Items

Six reviewers (J.K., S.K., A.K., M.L., M.M., and Z.M.) carried out the screening of the articles retrieved from the search. Each reviewer was assigned a portion of the articles and independently decided whether a given study was suitable for inclusion in the review. From the studies that met the eligibility criteria, data such as the first author, year of publication, study design, article title, fluoride release values, and type of varnish were extracted. All information was systematically recorded in a standardized Excel worksheet (Microsoft Excel 365, Version 2505, Build 16.0.18827.20102, 64-bit). To ensure consistency, the level of concordance was assessed using Cohen’s kappa statistic, and any disagreements regarding study inclusion were resolved through joint discussion between researchers until consensus was reached [53].

### 2.5. Protocol

**Figure 2:**
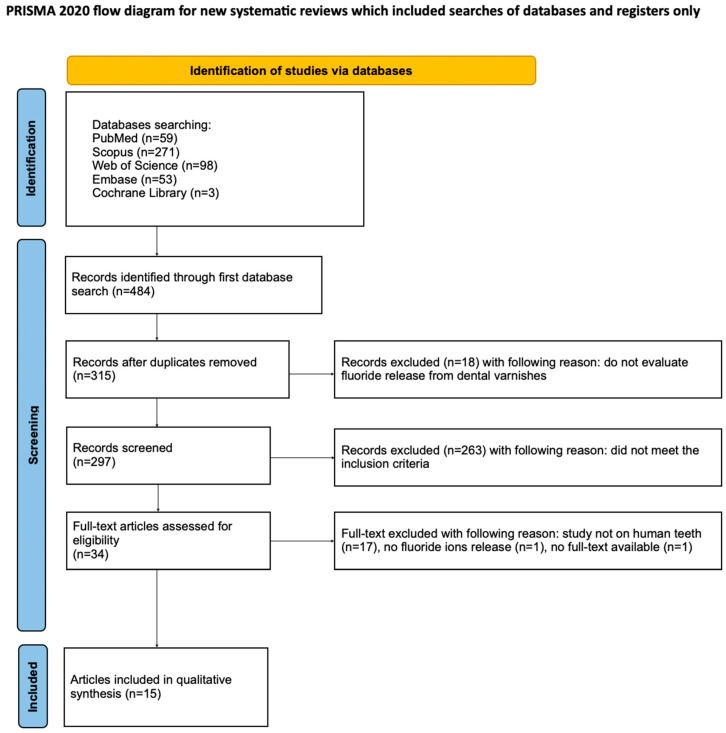
The PRISMA 2020 flow diagram [54].

The process of article selection for this systematic review was documented in accordance with the PRISMA flow diagram (Figure 2) [54]. The review was prospectively registered on the Open Science Framework at the following link: https://osf.io/z2hdr (accessed on 18 August 2025).

### 2.6. Risk of Bias and Quality Assessment

Two reviewers (J.M. and M.D.) independently and in a blinded manner evaluated the methodological quality of the included studies using the Joanna Briggs Institute (JBI) checklist for quasi-experimental (non-randomized) research [55]. Each checklist item was assessed separately, with possible responses categorized as “yes,” “no,” “unclear,” or “not applicable.” Any discrepancies were resolved through group discussion until consensus was reached. To provide an objective measure of inter-rater reliability, agreement between the two reviewers was quantified using Cohen’s kappa statistic, calculated with MedCalc software (version 23.1.7, MedCalc Software Ltd., Ostend, Belgium). The obtained kappa value was 0.89 (*p* < 0.001), indicating excellent agreement beyond chance.

## 3. Results

### 3.1. Study Selection

An initial search of PubMed, Scopus, Web of Science, Embase, and the Cochrane Library identified 484 potentially relevant records. After removing 168 duplicates, titles and abstracts were screened, and studies not involving in vitro research on human teeth with the application of fluoride varnish were excluded. Of the 35 articles retrieved for full-text evaluation, 20 did not meet the inclusion criteria [56,57,58,59,60,61,62,63,64,65,66,67,68,69,70,71,72,73] and were excluded. Ultimately, 15 studies were included in the qualitative synthesis [1,2,3,4,22,29,30,74,75,76,77,78,79,80,81]. Due to the substantial heterogeneity among the included studies, a meta-analysis could not be performed.

### 3.2. General Characteristics of the Included Studies

A total of fifteen studies met the inclusion criteria for this review [1,2,3,4,22,29,30,74,75,76,77,78,79,80,81]. Several of these studies investigated fluoride varnishes containing specific functional additives, as detailed in Table 2. Tricalcium phosphate (TCP) was incorporated in Clinpro White Varnish, Clinpro XT Varnish, β-TCP-F varnish, and Mahidol varnish, and was evaluated in eight studies [3,4,22,29,75,76,77,79]. Casein phosphopeptide–amorphous calcium phosphate (CPP–ACP) was present in MI Varnish [2,4,22,78]; in addition, study [3] examined a CPP–ACP-F paste, which did not meet the definition of a varnish. Amorphous calcium phosphate (ACP) was included in Enamel Pro and Premier Enamel Pro and assessed in five studies [4,22,74,79,80]. Xylitol-coated dicalcium phosphate dihydrate (DCPD) was present in Embrace Varnish and was evaluated in one study [22].

Fluoride release varied substantially depending on the varnish formulation and the experimental conditions applied. In most studies, peak release occurred within the first 24 h [1,2,4,74,75,76,78,79], followed by a marked decrease, whereas certain resin-modified varnishes and those supplemented with functional additives maintained ion release for longer periods [2,3,4,22,29,74,75,76,77,78,79]. Pańczyszyn et al. [78] demonstrated that acidic pH significantly enhanced fluoride release. Preparations containing remineralizing agents such as CPP–ACP promoted enamel rehardening and reduced demineralization [2,3,4], while bioactive additive–enriched varnishes showed antibacterial activity against *Streptococcus mutans* [2,3]. Other evaluated parameters included wettability and viscosity [2,15], fluoride recharge potential [75], surface hardness [3,4], and surface roughness [3]. Overall, varnish composition, environmental pH, and the incorporation of functional additives were the principal factors influencing both the extent and persistence of fluoride release, with advanced formulations offering added preventive effects (see Supplementary Table S1).

### 3.3. Main Study Outcomes

#### 3.3.1. Sample Size/Volume

Across the reviewed studies, the sample sizes and geometries varied notably, encompassing both whole teeth and prepared enamel or dentin specimens. Several investigations, such as those by Asian et al. [76], Thakur et al. [1], Rirattanapong et al. [79], Ritwik et al. [80], Castillo et al. [30], and Castillo et al. [81], used standardized small slabs or discs—most commonly 5 × 5 mm tooth blocks or discs of 10 mm in diameter and approximately 1 mm in thickness [77]. The studies used premolars, molars, and anterior teeth, either primary or permanent [1,2,3,4,22,29,30,74,75,76,77,78,79,80,81]. The number of specimens per group ranged widely—from as few as six in Okuyama et al. [77] disc-based protocol to over 120 teeth in Barrera-Ortega et al. [3] large-scale design. This variability in sample size, shape, and preparation method can influence surface area exposure and, consequently, fluoride release measurements, highlighting the importance of methodological consistency for cross-study comparisons.

#### 3.3.2. Storage Conditions

The reviewed publications presented substantial differences in storage protocols, encompassing the choice of medium, pH, temperature, and renewal frequency. Artificial saliva (AS) was the predominant storage medium, applied in 9 studies [1,4,22,29,74,78,79,80], generally at a near-neutral pH (7.0–7.2) and maintained either at room temperature or 37 °C, with regular replacement according to the study design. Buffered calcium phosphate solution appeared in three investigations [30,76,81], while deionized or distilled water was used by Okuyama et al. [77] and Nahum et al. [75] with daily or weekly renewal schedules. Barrera-Ortega et al. [3] stored samples in a demineralizing solution at pH 4.4 to replicate cariogenic conditions, whereas Pańczyszyn et al. [78] compared three pH levels (4, 5, and 7) to assess the influence of acidity. Several studies stored samples at 37 °C [2,3,4,75,77,78], while others used room temperature [1,29,30,74,79,80,81]. The storage medium volumes ranged from 3 mL [80] to 500 mL [1]. Most studies used volumes between 5 mL and 30 mL, with 20 mL [30,76,81] being one of the most common values. Such heterogeneity in storage parameters can significantly impact fluoride release rates and complicate direct comparison between studies.

#### 3.3.3. Measurement Methods and Time

Fluoride release in the included studies was quantified using various analytical techniques, with the ion-selective electrode (ISE) being the predominant choice [1,2,3,4,29,30,75,76,77,78,79,80,81], frequently combined with a TISAB III buffer to stabilize ionic strength and pH during measurements [4,29,30,77,79,80,81]. Specific equipment mentioned included Orion 9609 or the Versa Star A329 ion analyzer. Alternative approaches were less common—Singh et al. [74] used ion chromatography (Metrohm 940 Professional IC Vario, Metrohm AG, Herisau, Switzerland), while Sidhu et al. [22] employed the SPADNS spectrophotometric method at 570 nm. The duration of fluoride monitoring ranged from short-term protocols of 24–48 h [79,80] to extended observations lasting up to six months [22,29,74,81]. Many studies implemented intensive measurement schedules in the early phase, such as daily readings during the first week, followed by weekly or monthly intervals [2,30,76,81]. Some authors also performed post-recharge assessments to evaluate the capacity for secondary fluoride release after re-exposure to fluoride sources [75].

#### 3.3.4. Fluoride Release Results

Studies on fluoride release from dental varnishes demonstrated substantial variability in both initial concentrations and long-term release dynamics, depending on the product type and experimental conditions. Barrera-Ortega et al. [3] reported that the cumulative fluoride ions released over 15 days reached 72 ppm. Singh et al. [74] observed that Group III showed the highest fluoride release at most time points, with the peak value of 3.47 ppm on day 1, while at 6 months Group II demonstrated the highest level (0.16 ppm). Okuyama et al. [77] presented fluoride release profiles graphically, without precise numeric values available for extraction. Pańczyszyn et al. [78] confirmed a pH-dependent effect, with MI Varnish showing the highest release (up to 11.52 ppm) and Embrace Vanish the lowest (4.82 ppm). Yildiz et al. [4] observed peak values within the first 24 h for MI Varnish, while both Yildiz et al. [4] and Nahum et al. [75] reported high initial release for Duraphat and Clinpro White Varnish, followed by a marked decline. Asian et al. [76] confirmed the superiority of Duraphat (5.93 ppm) over other varnishes after 6 weeks, while Thakur et al. [1] reported the highest release for Bifluorid 10 (19.5 ppm) after 14 days. Sidhu et al. [22] found Clinpro White Varnish had the highest total release (20.26 mgF^−^/L), and Attiguppe et al. [2] showed MI Varnish releasing more fluoride than Fluor Protector at nearly all time points. Virupaxi et al. [29] demonstrated that Clinpro XT Varnish maintained the highest release at 6 months (9.78 ppm), outperforming Fluoritop SR and Fluorprotector. Rirattanapong et al. [79] noted that Duraphat and Clinpro White maintained higher long-term levels than Mahidol or Enamel Pro. Ritwik et al. [80] reported extremely high initial release for Premier Enamel Pro (1730.2 ppm) with a plateau after 4 h. Castillo et al. [30] showed greater release with three applications than a single one, while Castillo et al. [81] found that Duraphat released fluoride for up to 28 weeks compared with 19 weeks for Duraflor, with a more stable profile. Overall, most studies recorded the highest fluoride release within the first hours or days, followed by a significant decrease, although some varnishes (Clinpro XT, Duraphat) exhibited more stable, long-term release.

#### 3.3.5. Summary of Commercial Fluoride Varnishes

The included studies assessed a wide variety of commercial varnishes, differing in additives and performance profiles. First-generation formulations such as Duraphat and Fluor Protector demonstrated high initial fluoride release but generally showed a rapid decline over time and limited remineralization effects [1,2,3,4,22,29,30,74,75,76,78,79,80,81]. By contrast, newer varnishes enriched with bioactive additives provided distinct advantages. MI Varnish (CPP–ACP) consistently showed higher cumulative fluoride release, superior enamel microhardness recovery, and antibacterial activity [2,4,22,78]. Clinpro White Varnish (fTCP) and Clinpro XT (fTCP with resin base) exhibited stable, sustained release, with Clinpro XT demonstrating the most prolonged fluoride availability [22,29,75,76,77,78,79]. Enamel Pro (ACP) demonstrated the ability to reverse enamel demineralization and ranked among the top varnishes in early fluoride release [4,22,74,79,80]. Embrace Varnish (xylitol–calcium phosphate) released detectable fluoride but performed lowest overall in cumulative release [22,78]. Bifluorid 10 achieved very high short-term release [1], while Omni Vanish XT demonstrated an extended release profile compared with other resin-based formulations [80]. Duraflor exhibited a strong initial release but shorter duration compared with Duraphat [81]. Collectively, these findings suggest that bioactive additives (e.g., CPP–ACP, TCP, ACP) enhance the longevity and clinical relevance of fluoride release, offering potential advantages for preventive dentistry (see Table 1).

#### 3.3.6. Additional Findings

Additional findings from the included studies show that fluoride varnishes differ in effects beyond release rates. Barrera-Ortega et al. [3] found that β-TCP-F varnish and CPP-ACP-F paste helped reverse enamel changes from in vitro demineralization. Singh et al. [74] identified Enamel Pro as the top performer in fluoride release in artificial saliva for up to 3 months. Okuyama et al. [77] noted Clinpro XT released the most fluoride after 7 days. Pańczyszyn et al. [78] confirmed MI Varnish had the highest cumulative release, independent of pH, and Embrace Varnish the lowest. Yildiz et al. [4] reported MI Varnish achieved the best surface microhardness recovery. Nahum et al. [75] found Duraphat and Clinpro White Varnish released the most fluoride on day 1, with a marked drop after day 2. Asian et al. [76] reported Duraphat’s highest release over 6 weeks, linked to high viscosity and low wettability. Thakur et al. [1] recorded the highest 14-day release for Bifluorid 10. According to Sidhu et al. [22], Clinpro White Varnish demonstrated the greatest overall fluoride release within a 6-month period, MI Varnish showed the longest-lasting fluoride retention, while Embrace Varnish exhibited the quickest reduction in available fluoride. Attiguppe et al. [2] showed MI Varnish outperformed Fluor Protector in release, remineralization, and antibacterial activity. Virupaxi et al. [29] observed Clinpro XT’s stable, sustained release; Rirattanapong et al. [79] did not assess biological effects. Ritwik et al. [80] found Premier Enamel Pro led in the first 8 h, while Omni Vanish XT had the best sustained release after 4 h. Castillo et al. [30] reported 47% more total release with three applications. In the study by Castillo et al. [81], Duraflor exhibited a greater initial rate of fluoride release, while Duraphat surpassed it in the later phase, with both products showing comparable physical characteristics (see Table 2).

### 3.4. Quality Assessment of Individual Studies

**Figure 3:**
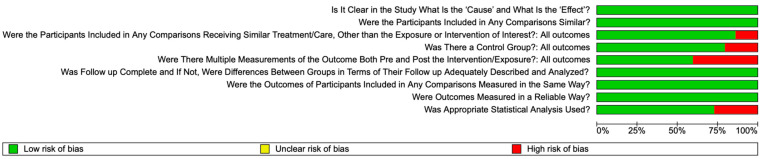
Risk of bias of included studies.

Out of the nine quality assessment items, eight studies achieved eight positive answers [1,3,22,29,74,76,77,79]. Five studies obtained seven positive responses [4,30,78,80,81], while two studies received six positive answers [2,75]. This distribution demonstrates that most included studies exhibited strong methodological quality, with only minor limitations identified in a small number of cases. The high risk of bias arises from the fact that the included studies reported only post-exposure measurements, which led to the assessment being marked in red on the risk-of-bias graph (see Figure 3, and Supplementary Table S2).

## 4. Discussion

The present review confirms that the chemical composition of fluoride varnishes is a decisive factor for ion release, which is consistent with previous observations reported by Singh et al. [74], Yildiz et al. [4], and Sidhu et al. [22]. In particular, formulations supplemented with calcium- and phosphate-based compounds such as CPP–ACP, TCP, or ACP have repeatedly demonstrated higher and more sustained release than conventional formulations [82,83]. This can be explained by the additional ion reservoirs provided by these additives, which prolong fluoride availability and enhance the potential for remineralization. Similar conclusions were drawn by Pańczyszyn et al. [78], who confirmed a pH-dependent increase in fluoride release, while other authors [84,85,86,87] emphasized that acidic conditions intensify ion liberation—a phenomenon of particular clinical value for patients exposed to frequent cariogenic challenges. The importance of the carrier system has also been noted: varnishes incorporating a glass-ionomer matrix maintained release for up to six months, echoing earlier reports of a reservoir effect with potential long-term preventive benefits for patients at high caries risk. By contrast, formulations without bioactive components exhibited a rapid decline in fluoride release over time [22,29,33,74]. It should also be noted that methodological variability across studies—differences in sample geometry, surface standardization, or storage conditions [1,2,3,4,22,74,75,77,78,79,80,81]- limits direct comparison. Nevertheless, taken together, the available evidence underscored the superiority of bioactive formulations and provided a mechanistic explanation for their more favourable performance in caries prevention.

The diversity across the included studies, even when testing the same varnish, offers valuable insights into the optimal application of these products to maximize their preventive benefits. The reviewed evidence confirms that fluoride release from varnishes typically follows a biphasic pattern, with an initial burst of ion liberation followed by a gradual decline [83,88]. This early peak, most pronounced within the first 24 h, has been consistently reported across multiple studies [2,4,74,75], and reflects the release of loosely bound fluoride at the varnish–tooth interface. Alternative patterns have also been described: Sidhu et al. [22] observed a delayed maximum release between the first and third month, while Virupaxi et al. [29] recorded the highest release after one week. Such variability underscores the importance of material composition and matrix design. Acidic pH conditions were shown to significantly increase ion release [78,84,89], suggesting that varnishes may provide enhanced protection under cariogenic challenges when additional fluoride is most needed. Fluoride concentration itself is another determinant, as formulations with higher baseline fluoride (e.g., 22,600 ppm F) demonstrated several-fold greater cumulative release compared with those containing lower concentrations (e.g., 1000 ppm) [75,76]. Nevertheless, concentration alone does not explain all differences: products with similar NaF content exhibited divergent profiles, as exemplified by Omni Vanish XT and Duraflor, both containing 5% NaF yet showing markedly different release dynamics [80,81]. These discrepancies highlight the role of the carrier system and the inclusion of functional additives. Varnishes supplemented with calcium- and phosphate-based compounds, such as CPP–ACP or functionalized tricalcium phosphate, consistently showed more sustained release and superior preventive potential compared with fluoride-only formulations [85,86,87,90,91,92,93]. This aligns with broader evidence that bioactive additives not only prolong fluoride availability but also enhance remineralization and antibacterial properties, thereby explaining the superiority of newly developed varnishes in preventive dentistry.

Beyond the absolute amount of fluoride released, several additional properties contribute to the clinical effectiveness of varnishes. Their remineralization potential has been consistently demonstrated [2,3,94,95], and is strongly linked to the inclusion of calcium–phosphate phases such as β-TCP, which provide an additional ion reservoir and facilitate the repair of demineralized enamel. Improvements in enamel microhardness have also been observed [4,96], indicating that varnishes can not only reverse early caries but also enhance the resistance of sound tissues to future demineralization [97,98]. Antibacterial activity has been reported as well, suggesting a broader preventive effect that extends beyond remineralization. Physical parameters such as viscosity, wettability, and the number of applications further modulate fluoride dynamics. Repeated or sequential applications were shown to prolong fluoride ion availability and thereby sustain the anticaries effect [30,76,99,100,101]. From a clinical perspective, this strategy may be particularly relevant in high-risk patients, for whom extended fluoride exposure is essential. Another practical consideration is the combined use of varnishes with other fluoride sources. While high-concentration varnishes (e.g., 22,600 ppm NaF) rapidly elevate intraoral fluoride levels [75,76,79], their preventive effect is maximized when complemented by daily use of fluoride toothpaste or rinses. In this context, extended-release formulations can provide long-term baseline protection, whereas conventional sodium fluoride varnishes may require more frequent application to ensure continuous efficacy [30,99,100,101].

An important clinical question is whether new-generation fluoride varnishes provide additional benefits in caries prevention compared with first-generation products. MI Varnish, enriched with CPP–ACP, demonstrated higher cumulative fluoride release [78], superior recovery of enamel microhardness [4], and notable antibacterial activity [2] relative to conventional varnishes such as Fluor Protector or Duraphat. Enamel Pro Varnish, supplemented with ACP, showed the capacity to reverse enamel demineralization in vitro and ranked among the top performers in terms of fluoride release [4,74,80]. These findings suggest that newer formulations not only extend fluoride availability but also enhance remineralization and, in some cases, provide antibacterial effects, offering distinct advantages for preventive dentistry in clinical practice. From a clinical perspective, these properties may be particularly relevant under conditions of high cariogenic challenge, where sustained fluoride release and the synergistic action of bioactive additives can help counteract continuous acid attacks. Furthermore, it should be considered that varnishes are often used in combination with other fluoride-containing products, such as toothpastes and mouthrinses, which may influence fluoride bioavailability and overall preventive efficacy. Future research should therefore focus on evaluating how new-generation varnishes perform in real-world scenarios that include simultaneous exposure to multiple fluoride sources and variable cariogenic risk profiles.

Several limitations should be considered when interpreting the findings of this review. First, all included studies were conducted in vitro, which restricts the direct translation of results to clinical practice. In addition, there was considerable methodological diversity across studies, including differences in the type of teeth used (permanent vs. primary), the immersion medium (artificial saliva vs. deionized water), and the timing of measurements, which ranged from 30 min to 6 months. Another limitation is the narrow scope of outcomes assessed: most investigations focused exclusively on quantifying fluoride ion release into solution, while only a few (e.g., Yildiz et al., Attiguppe et al.) examined additional parameters such as surface microhardness or antibacterial activity [2,4]. Finally, this review was limited to English-language publications, which may have introduced language bias and excluded relevant studies reported in other languages.

## 5. Conclusions

In summary, this review demonstrates that fluoride release from varnishes is a multifactorial process, primarily determined by the chemical composition of the product, the surrounding pH, and the time elapsed since application. The greatest clinical benefits are offered by formulations that combine fluoride with additional calcium- and phosphate-based components (e.g., MI Varnish or Enamel Pro) or by glass ionomer-based varnishes (e.g., Clinpro XT), which provide a more sustained release profile. In contrast, varnishes characterized by low viscosity and lacking mineral additives (e.g., Fluor Protector) appear less effective. Although these in vitro results are promising, further high-quality clinical trials are necessary to validate their effectiveness under intraoral conditions and to establish optimal protocols for caries prevention in different age groups and patient populations with varying caries risk.

## Figures and Tables

**Table 1:** Commercial fluoride varnishes, composition, and main findings from the included studies.

Fluoride Varnish	Additive(s)	Main Positive Findings	Main Limitations/Negatives	References
Duraphat	NaF (5%), no additive	Sustained release (up to 28 weeks), widely studied, clinical benchmark	Rapid decline after initial release, less effect on remineralization vs. bioactive formulations	[30,75,76,78,79,81]
Fluor Protector	Amine fluoride	Stable compound, moderate release	Lowest cumulative release in several studies, limited remineralization	[1,2,4,29,75,76]
Clinpro White Varnish	fTCP	High cumulative release (20.26 mg/L), sustained over months	Initial burst followed by decline, variable performance	[22,75,76,78,79]
Clinpro XT	fTCP + resin-modified base	Most stable, long-term release, good substantivity	Requires light-curing, less studied clinically	[29,77]
MI Varnish	CPP–ACP	High cumulative release, superior microhardness recovery, antibacterial effect	Performance depends on medium pH, limited long-term data	[2,4,78]
Enamel Pro	ACP	High fluoride release, ability to reverse demineralization	Lower long-term release vs. TCP products	[4,22,74,79,80]
Embrace Varnish	Xylitol–calcium phosphate	Fluoride release demonstrated	Lowest cumulative release among tested products	[22,78]
Bifluorid 10	NaF + CaF_2_	Very high release in first 14 days	Short-term effect, limited long-term data	[1]
Omni Vanish XT	NaF (resin-based)	Sustained release beyond 48 h, extended profile	Limited independent studies	[80]
Duraflor	NaF	High initial release, widely used	Release stops earlier than Duraphat (~19 weeks)	[81]
Other formulations (Mahidol, Varnal, etc.)	TCP/experimental bases	Variable effects, some strong initial release	Limited replication, early-stage data	[76,79]

**Table 2:** Detailed characteristics of included studies.

Authors	Study/Samples Design	Fluoride Varnish	Storage Conditions	Measurement Time and Method	Total Fluoride Released	Additional Findings
Barrera-Ortega [3]	In vitro, 120 human third molars	- β-TCP-F (ClinproTM White Varnish, St. Paul, MN, USA)	Demineralizing solution (2.2 mM CaCl_2_, 2.2 mM NaH_2_PO_4_, and 0.05 M CH_3_COOH) for 96 h at 37 °C, pH 4.4	5, 10, 15 days Method: 10 surfaces of each group were randomly chosen and immersed in deionized water at 37 °C Fluoride-Ion Selective Electrode (F-ISE) (Orion Star A-214)	After 15 days CPP-ACP-F showed higher fluoride concentrations (ppm) in remineralizing solutions than β-TCP-F.	β-TCP-F varnish and CPP-ACP-F paste treatments demonstrated the ability to counteract surface modifications on human enamel caused by in vitro demineralization.
Singh [74]	In vitro 72 healthy permanent maxillary anterior teeth.	- Fluor Protector (Ivoclar Vivadent, New York, USA) - Enamelast varnish (Ultradent Products, Cologne, Germany) - Enamel Pro varnish (Premier Dental, Pennsylvania, USA) - control group	In artificial saliva with pH 7.2 at room temperature.	1, 3, and 6 months Metrohm 940 Professional IC Vario	Fluoride release (ppm) Day 1 Group I 0.56 ± 0.09 Group II 1.38 ± 0.21 Group III 3.47 ± 0.19 1 month Group I 0.36 ± 0.06 Group II 1.24 ± 0.13 Group III 1.57 ± 0.12 3 months Group I 0.11 ± 0.02 Group II 0.26 ± 0.06 Group III 0.32 ± 0.08 6 months Group I 0.03 ± 0.01 Group II 0.16 ± 0.06 Group III 0.09 ± 0.03	Enamel Pro released the maximum amount of fluoride in artificial saliva for up to 3 months.
Okuyama [77]	In vitro, 6 disc- shaped specimens (10 × 1 mm)	- PRG Barrier Coat (Shofu, Kyoto, Japan), - Clinpro XT varnish (3M, Minnestota, USA), - Fuji IX EXTRA (GC, Japan), - Clearfil Mega Bond (Kuraray Noritake Dental, Osaka, Japan)	- 8 mL of deionized water - 37 °C - distilled water changed every day for 7 days and weekly up to 28 days	-TISAB III -Fluoride-selective electrode	After 7 days Clinpro XT varnish ~200 μg/cm^2^, followed by Fuji IX EXTRA, PRG Barrier Coat and Clearfil Mega Bond. Clinpro XT varnish: release increased over time reaching ~380 μg/cm^2^	After 7 days no significant difference in fluoride release between the Fuji IX EXTRA and PRG Barrier Coat groups. Clinpro XT varnish released the most fluoride, whereas Clearfil Mega Bond released the least.
Pańczyszyn [78]	In vitro, 45 human molars, free from caries, demineralization, and enamel defects	- Duraphat (Colgate Oral Care, Sydney, NSW, Australia), - MI Varnish (GC, Tokyo, Japan), - Embrace Varnish (Pulpdent, Watertown, MA, USA)	- 5 mL of artificial saliva (NaCl, KCl, urea, Na_2_S·9H_2_O, NaH_2_PO_4_·2H_2_O, CaCl_2_·2H_2_O) - pH = 4; 5; 7 - 37 °C	-measurement after 1, 2, 24, 48 and 168 h -ORION 9609 Model ion selective electrode with the CPI-551 Elmetron microcomputer	(a) Duraphat pH = 4: 9.753 ppm pH = 5: 7.513 ppm pH = 7: 9.276 ppm (b) MI Varnish pH = 4: 11.52 ppm pH = 5: 9.297 ppm pH = 7: 6.470 ppm (c) Embrace Vanish: pH = 4: 6.826 ppm pH = 5: 5.724 ppm pH = 7: 4.821 ppm	MI Varnish demonstrated the highest cumulative fluoride release, independent of the environmental pH; Embrace Varnish exhibited the lowest fluoride release.
Yildiz [4]	In vitro, 48 caries- free human molars	-MI Varnish (GC, America, USA) -Clinpro White Varnish (3M ESPE, MN, USA) -Duraphat (Colgate-Palmolive, NSW, Australia) -Fluor Protector (Ivoclar Vivadent, NY, USA) -Enamel Pro (Premier Dental, PA, USA)	- 10 mL of artificial saliva - 37 °C - Sample incubated for 2, 24, 48 h, and 7 days, with daily saliva renewal. After 7 days, they were rinsed, the fluoride varnish was removed and incubated again in fresh saliva for 24 h.	-TISAB III buffer added to the solution -Ion-selective electrode	MI Varnish: -2 h: 6.72 (3.44) ppm -24 h: 22.66 (6.79) ppm -48 h: 0.76 (0.26) ppm -7 days: 0.084 (0.11) ppm Clinpro White Varnish: -2 h: 0.62 (0.27) ppm -24 h: 5.07 (3.87) ppm -48 h: 2.22 (0.85) ppm -7 days: 0.48 (0.16) ppm Duraphat: -2 h: 2.3 ± 0.54 ppm -24 h: 12.81 ± 4.85 ppm -48 h: 2.70 ± 0.90 ppm -7 days: 0.69 ± 0.31 ppm Fluor Protector: -2 h: 0.37 ± 0.096 ppm -24 h: 0.42 ± 0.15 ppm -48 h: 0.05 ± 0.03 ppm -7 days: 0.05 ± 0.03 ppm Enamel Pro Varnish: -2 h: 2.33 ± 0.94 -24 h: 12.30 ± 5.10 -48 h: 5.52 ± 2.64 -7 days: 0.62 ± 0.31	MI Varnish showed the highest surface microhardness recovery. All varnishes significantly improved enamel microhardness compared to the control. No significant differences were found between varnishes, except MI Varnish performed better than Fluor Protector.
Nahum [75]	In vitro, 40 human premolars and molars; rectangular blocks	-Duraphat 2.26% (Colgate Palmolive, New York, USA) -Clinpro White Varnish 2.26% (3M ESPE, Minnesota, USA) -Fluor Protector 0.1% (Ivoclar-Vivadent, Schaan, Liechtenstein) Single application.	In plastic bottles, 5 ml deionized water, 37 °C; On each measurement day, the sample was rinsed with 1 mL of deionized water before being placed in a new container.	-ion selective electrode for sodium fluoride (model 1011, Hanna Instruments, USA) and potentiometer (model HI 3222, Hanna Instruments); measured in: 1, 2, 5, 15 and 30 days. After recharging measured in 24, 48, 72 h	-Duraphat—9.51 ppm -Clinpro White Varnish—10.16 ppm -Fluor Protector—5.01 ppm After recharging: -Duraphat—3.0 ppm -Clinpro White Varnish—3.02 ppm -Fluor Protector—3.0 ppm	DP and CWV released the highest amount of fluoride on day 1 and throughout the study.
Asian [76]	In vitro, 44 enamel blocks 5 × 5 mm from human premolars	-Duraphat (Colgate-Palmolive, New York, NY, USA) -ClinproTM White Varnish (3M ESPE, Minnesota, USA) -Fluor Protector (Ivoclar Vivadent, Amherst, New York, USA) -Varnal (Biodinámica, Paraná, Brasil)-control Single application: 30 mg of fluoride varnish; DP and Clinpro-37.5 μmol of fluoride; Fluor Protector 1.58 μmol of fluoride	20 mL-buffer calcium phosphate solution; pH = 6.0; temperature 5 °C	-ion analyzer (Versa Star A329, Orion, Ther- mo Scientific) and a fluoride selective electrode (Plus Model 9606 VPN, Orion, Thermo Scientific) Measurements taken daily in the first week, once a week for the remaining six weeks.	For 6 weeks: -Duraphat—5.9266 ppm -ClinproTM White Varnish—2.2148 ppm -Fluor Protector—0.407 ppm -Varnal control—0.21196 ppm	Duraphat released the highest amount of fluoride over 6 weeks. The highest viscosity and the lowest wettability- Duraphat The higher the viscosity and lower the wettability, the better the varnish’s ability to release fluoride.
Thakur [1]	In vitro, 96 human premolars	-38% Silver Diamine Fluoride (e-SDF, India) -Bifluorid 10 (Voco, Germany) -1.23% Acidulated phosphate fluoride gel Fluocal Gel (Septodont, France) -control sample (no varnish)	In plastic containers, artificial saliva, 500 mL, pH = 7, room temperature,	Ion-sensitive electrode Measurement after: -24 h, -7 days -14 days	For 14 days: -38% Silver Diamine Fluoride: 9.06 ppm -Bifluorid 10: 19.5 ppm -1.23% Acidulated phosphate fluoride gel Fluocal Gel: 3.42 ppm -control sample (no varnish): 0.64 ppm	Varnish Bifluorid released highest amount of fluoride for 14 days.
Sidhu [22]	In vitro 75 extracted, caries-free premolar human teeth	Type: -MI Varnish (CPP-ACP) (GC, Tokyo, Japan) -Clinpro White Varnish (f-TCP) (3M ESPE, Minnesota, USA) -Embrace Varnish (Xylitol-coated calcium and phosphate) (Pulpdent, Watertown, MA, USA) -Enamel Pro Varnish (ACP) (Premier Dental, Pennsylvania, USA) Fluoride concentration: 5% NaF Application protocol: Applied varnish to a 3 × 3 mm window on the tooth surface; immersed in 50 mL artificial saliva.	- 50 mL of artificial saliva in plastic containers; replaced at specific intervals.	Time points: -1 day, -1 month, -3 months, -6 months. SPADNS method with a spectrophotometer (570 nm).	MI Varnish 1 Day: 1.52 mgF^−^/L 1 Month: 6.45 mgF^−^/L 3 Months: 6.95 mgF^−^/L 6 Months: 3.50 mgF^−^/L Total: 18.42 mgF^−^/L Clinpro White Varnish 1 Day: 1.76 mgF^−^/L 1 Month: 7.60 mgF^−^/L 3 Months: 8.05 mgF^−^/L 6 Months: 2.85 mgF^−^/L Total: 20.26 mgF^−^/L Embrace Varnish 1 Day: 1.52 mgF^−^/L 1 Month: 6.71 mgF^−^/L 3 Months: 6.55 mgF^−^/L 6 Months: 1.95 mgF^−^/L Total: 16.73 mgF^−^/L Enamel Pro Varnish 1 Day: 1.35 mgF^−^/L 1 Month: 7.20 mgF^−^/L 3 Months: 7.30 mgF^−^/L 6 Months: 2.95 mgF^−^/L Total: 18.50 mgF^−^/L	Clinpro White Varnish: highest cumulative fluoride release over 6 months (20.26 mgF^−^/L). MI Varnis: highest substantivity, released the most fluoride by 6 months (3.5 mgF^−^/L at 6-month mark). Embrace Varnish: lowest cumulative release and fastest depletion.
Attiguppe [2]	In vitro 24 extracted human premolar teeth	-MI Varnish: 5% NaF + CPP–ACP (GC, Tokyo, Japan) -Fluor Protector (Ivoclar Vivadent, Amherst, New York, USA) Application: varnish and fluor protector varnish was applied on 5 mm × 1 mm surface	- 30 mL of artificial saliva at 37 °C; - the saliva was replaced at each time point	Fluoride ion-selective electrode measurements: -after 30 min, -daily for the first 7 days -weekly up to 1 month	Cumulative fluoride release: -MI varnish: 4.19 ± 0.41 ppm -Fluor Protector: 3.2 ± 0.19 ppm	MI varnish released more fluoride than Fluor Protector. MI varnish resulted in lower lesion depth (79.78 μm vs. 119.2 μm), showing better demineralization resistance. MI varnish showed a larger inhibition zone against Streptococcus mutans (24.75 mm vs. 15.25 mm).
Virupaxi [29]	In vitro, 24 extracted human primary anterior teeth	- Clinpro XT Varnish (3M ESPE, Minnesota, USA); - Fluoritop SR (ICPA, Mumbai, India); - Fluor Protector (Ivoclar Vivadent, Amherst, New York, USA) Application protocol: Teeth coated with varnishes 3 × 3 mm window. Fluorprotector and Fluoritop SR applied using the supplied brush, while Clinpro XT was mixed as directed and light-cured for 20 s.	Artificial saliva (pH 7.2) at room temperature; medium renewed at: 1 day, 1 month, 3 months, and 6 months	Ion-selective electrode (ISE) with TISAB III buffer Fluoride concentration measured at 1 week, 1 month, 3 months, and 6 months	Clinpro XT Varnish 9.78 ± 4.11 ppm Fluoritop SR 0.61 ± 0.36 ppm Fluorprotector 0.17 ± 0.02 ppm	Clinpro XT demonstrated the most stable and sustained fluoride release compared to Fluoritop SR and Fluorprotector; favourable profile for long-term remineralization due to glass ionomer base.
Rirattanapong [79]	In vitro, 25 extracted sound human primary incisors	- Duraphat: (5%NaF) (Colgate Oral Pharmaceuticals New York, NY, USA) - Clinpro White: (5%NaF) + (TCP) (Premier Dental, Hannover, Germany) - Enamel Pro: (5%NaF) + (ACP) (3M ESPE, West Palm Beach, FL, USA) - Mahidol varnish: (5%NaF) + (TCP) (Mahidol University, Thailand) Application: Approximately 30 mg of the assigned fluoride varnish was applied to each prepared tooth (5 × 5 mm window).	- 60 mL of artificial saliva at room temperature; - maintained on a laboratory shaker to simulate oral conditions	Fluoride ion-selective electrode (Orion 96-09) with TISAB III Fluoride release assessed at 2, 4, 8, 12, 24, and 48 h, then weekly for 3 months	Duraphat: 11.42 ± 0.67 Clinpro White: 11.19 ± 0.38 Enamel Pro: 3.72 ± 0.27 Mahidol varnish: 8.36 ± 0.41	All varnishes released significantly more fluoride than control. Mahidol varnish had the highest release in the first 24 h (0.87 ppm). After 3 months, fluoride release order: Duraphat = Clinpro White > Mahidol > Enamel Pro > Control. Duraphat showed lower initial release but more sustained levels over time.
Ritwik [80]	In vitro study, 50 extracted permanent human teeth divided into 5 groups (n = 10). Varnish application (5 × 5 mm window).	Single application, all containing 5% NaF (22,600 ppm): - Enamel Pro Varnish (EP), -Colgate PreviDent (CP), -Omni Vanish (OV), -Omni Vanish XT (OVXT).	Medium: 3 mL artificial saliva, pH 7.2, room temperature	Time points: 1, 2, 4, 8, 12, 24, and 48 h Measurements with ion selective electrode with TISAB III. Duration: 48 h	At 1 h: -Premier Enamel Pro: 1730.2 ppm (highest initial release) -Omni Vanish XT: 487.1 ppm -Colgate PreviDent: 163.5 ppm -Omni Vanish: 45.8 ppm Mean hourly release rate: -Premier Enamel Pro: 358.467 ppm/hour (±124.712) -Omni Vanish XT: 188.676 ppm/hour (±106.484) -Colgate PreviDent: 52.244 ppm/hour (±10.081) -Omni Vanish: 18.470 ppm/hour (±5.959)	EP had highest fluoride release in first 8 h. OVXT had highest sustained release after initial 4 h period. All varnishes showed significantly different fluoride release profiles (*p* < 0.0001).
Castillo [30]	In vitro study, 14 primary molar slabs (5 × 5 mm) from exfoliated teeth collected in nonfluorinated water communities (Lima, Peru), divided into three groups: 5 samples single application, 5 samples three applications, 4 unpainted controls. Slabs painted with 30 mg fluoride varnish.	Duraphat (Colgate-Palmolive, New York, USA)) 5% NaF (2.26% fluoride, equivalent to 35.7 micromoles fluoride per 30 mg application). 1: one application at baseline 3: at baseline, day 2, day 4 in one week	Medium: 20 mL buffered calcium phosphate solution (1.5 mM calcium nitrate, 1.0 mM sodium phosphate monobasic, 0.35 mM MES buffer), pH 6.0, room temperature	Duration: 6 months (21 weeks—stopped due to fungal growth). Weekly measurements with ion selective electrode with TISAB solution (low-level TISAB and TISAB III)	Total release (21 weeks): -single application: 23.7 ± 1.6 μmol (64.9% of applied fluoride released) -three applications: 34.9 ± 0.3 μmol (31.9% of applied fluoride released)	Three-application protocol showed 47% higher total fluoride release compared to single one. Three-application samples released more fluoride in weeks 8–21 and had slower release rate indicating longer availability of fluoride.
Castillo [81]	In vitro study, 23 primary molar slabs (5 × 5 mm) from exfoliated teeth collected in nonfluorinated water communities (Lima, Peru), divided into three groups: 9 samples Duraphat, 9 samples Duraflor, 5 untreated controls. Slabs painted with 30 mg fluoride varnish	-Duraphat 5% NaF (Colgate-Palmolive Co., New York, USA) -Duraflor 5% NaF (Pharmascience Inc., Montreal, Canada)) Protocol: single application with varnish from 9 different tubes of each product to assess inter-tube variability.	Medium: 20 mL buffered calcium phosphate solution, pH 6.0, room temperature.	Duration: 6 months (24 weeks). Weekly measurements with ion selective electrode with TISAB	Total Release over 24 weeks: -Duraphat: 25.1 ± 4.9 μmol (67% of applied fluoride released) -Duraflor: 20.2 ± 14.7 μmol (56% of applied fluoride released)	Initial 3 weeks: Duraflor had higher release rate but from week 4 onwards Duraphat had higher release rate. Weeks 4–24: no difference in release rates between products (*p* < 0.18) Physical prosperities: -Duraphat: more viscous, dries faster -Duraflor: less viscous Duraphat released fluoride until week 28 while Duraflor until week 19.

## Data Availability

No new data were created or analyzed in this study. Data sharing is not applicable to this article.

## Supplementary Materials

The following supporting information can be downloaded at: https://www.mdpi.com/article/10.3390/ma18194603/s1, Table S1: General Characteristics of Included Studies; Table S2: Quality Assessment of Included Studies.
