# Supplementary material for: Evaluation of Factors Affecting Fluoride Release from Fluoride Varnishes: A Systematic Review
Source: Materials (Basel). 2025 Oct 4;18(19):4603. doi: 10.3390/ma18194603 (PMC12525796; doi:10.3390/ma18194603)
Supplement: Supplementary file 1 [file materials-18-04603-s001.zip › materials-3865383-supplementary.pdf]

Supplementary Materials

Evaluation of Factors Affecting Fluoride Release from Fluoride Varnishes: A Systematic Review

Maciej Dobrzyński [1,*], Agnieszka Kotela [2], Sylwia Klimas [1], Zuzanna Majchrzak [2], Julia Kensy [3], Marzena Laszczyńska [2], Mateusz Michalak [2], Zbigniew Rybak [4], Magdalena Fast [5] and Jacek Matys [6,*]

[1] Department of Pediatric Dentistry and Preclinical Dentistry, Wroclaw Medical University, Krakowska 26, 50-425 Wroclaw, Poland; sylwia.klimas@student.umw.edu.pl

[2] Medical Center of Innovation, Wroclaw Medical University, Krakowska 26, 50-425 Wroclaw, Poland; kotela.agnieszka@gmail.com (A.K.); zuzanna.h.nawrocka@gmail.com (Z.M.); marzenalaszczynska@gmail.com (M.L.); mateusz.michalak92@gmail.com (M.M.)

[3] Faculty of Dentistry, Wroclaw Medical University, Krakowska 26, 50-425 Wroclaw, Poland; julia.kensy@student.umw.edu.pl

[4] Pre-Clinical Research Centre, Wroclaw Medical University, Bujwida 44, 50-345 Wroclaw, Poland; zbigniew.rybak@umw.edu.pl

[5] Department of Drug Form Technology, Wroclaw Medical University, Borowska 211 A, 50-556 Wroclaw, Poland; magdalena.fast@umw.edu.pl

[6] Dental Surgery Department, Wroclaw Medical University, Krakowska 26, 50-425 Wroclaw, Poland

* Correspondence: maciej.dobrzynski@umw.edu.pl (M.D.); jacek.matys@umw.edu.pl (J.M.)

Table S1. General Characteristics of the Included Studies.

Authors	Aim of the study	Material and Methods	Results	Conclusions
Barrera-Ortega [3]	In vitro comparison of protective effect of β-TCP-F varnish and CPP-ACP-F paste on artificially demineralized enamel	A total of 120 third molars were randomly allocated into four groups (n = 30 each):<br><br>**Group I:** Control (healthy enamel)<br>**Group II:** Initially demineralized enamel with lesions<br>**Group III:** Demineralized enamel treated with varnish<br>**Group IV:** Demineralized enamel treated with paste<br>The 15-day study involved daily cycles of 21 h demineralization (pH 4.4) and 3 h remineralization (pH 7), with varnish or paste applied to Groups III and IV before each cycle. Fluoride ion release was measured after every cycle, while surface hardness, roughness, wettability, and *Streptococcus mutans* biofilm formation were assessed on days 5, 10, and 15.	Treatment with CPP-ACP-F led to a greater increase in surface hardness compared to β-TCP-F . Both treatments reduced surface roughness relative to initially lesioned enamel.. Wettability improved in both groups, with contact angles of 71.01° (β-TCP-F) and 65.24° (CPP-ACP-F), approaching or surpassing values for healthy enamel.	Both β-TCP-F and CPP-ACP-F treatments showed protective effects against enamel demineralization. However, CPP-ACP-F demonstrated superior results, leading to greater surface hardness and a smoother enamel texture under in vitro pH cycling conditions. Both treatments effectively helped surface damage caused by demineralization on healthy human enamel.
Singh [74]	Comparison of the duration of fluoride release from various varnishes (Fluor Protector, Enamelast, Enamel Pro).	Materials: 72 healthy permanent maxillary anterior teeth.<br>Method: All tooth surfaces were coated with colored nail varnish specific to each group, leaving a 3 × 3 mm window on the labial surface for material application.<br>Group I: Fluor Protector varnish<br>Group II: Enamelast varnish	Fluor protector released the least fluoride over time . Enamelast demonstrated consistent fluoride release over six months. Enamel Pro showed the highest fluoride release up to 3 months, but its release decreased by 6 months.	Based on fluoride release over a six-month period, Enamel Pro varnish demonstrated the highest efficacy, followed by Enamelast and Fluor Protector. Enamel Pro is the most efficient in long term caries prevention.

		Group III: Enamel Pro varnish Group IV (Control): No material applied All groups were stored in artificial saliva (pH 7.2) at room temperature. After 24 hours, specimens were transferred to fresh containers with new artificial saliva, and the previous solutions were collected for fluoride analysis. This procedure was repeated to assess fluoride release (in ppm) at 1, 3, and 6 months.		
Okuyama [77]	To assess how much fluoride binds to dentin after applying different fluoride-releasing tooth coatings, using an in-air micro-PIXE/PIGE system.	48 extracted human molars were used. Root dentin surfaces were treated with one of four materials such as: PRG Barrier Coat, Clinpro XT varnish, Fuji IX EXTRA, and a non-fluoride control-Clearfil Mega Bond. Samples were immersed in a remineralizing solution for 7 or 28 days. Fluoride distribution in dentin was analyzed using in-air micro-PIXE/PIGE. Fluoride uptake in hydroxyapatite and fluoride release from the materials were measured using a fluoride electrode.	Fluoride uptake was highest at the dentin-material interface and declined within the first 100 μm. Clinpro XT varnish released the most fluoride but had the lowest proportion of tooth-bound fluoride (T-F), especially after 7 days. Fuji IX EXTRA and PRG Barrier Coat showed moderate fluoride release with more consistent T-F/W-F ratios. Clearfil Mega Bond, had the lowest fluoride values. In fluoride-treated hydroxyapatite, both water-soluble (W-F) and tooth-bound fluoride increased with solution concentration. Over time,	Fluoride-releasing coatings and varnishes increase both total and water-soluble fluoride in dentin, while those releasing less fluoride result in a higher proportion of tooth-bound fluoride.

			the T-F/W-F ratio varied depending on fluoride concentration and exposure duration.	
Pańczyszyn [78]	To examine how different fluoride varnishes release fluoride over time in various pH conditions.	45 extracted human premolars and molars were coated with one of three fluoride varnishes: Duraphat (first-generation), MI Varnish, or Enamel Varnish (second-generation). Teeth were immersed in artificial saliva at pH 4.0, 5.0, or 7.0 and stored at 37 °C. Fluoride release was measured at multiple time points (1–168 h) using an ion-selective electrode.	MI Varnish released the highest amount of fluoride across all pH levels, while Embrace Varnish released the least. Fluoride release was greater in more acidic environments and occurred most rapidly within the first 2 hours for all varnishes. MI and Embrace Varnish released over 90% of their total fluoride within 24 hours, while Duraphat showed slower, more prolonged release. Fluoride release followed an exponential decline over time.	Fluoride release from varnishes is strongly influenced by varnish type, time, and pH, with the highest release occurring shortly after application.
Yildiz [4]	To investigate the effectiveness of different fluoride varnish formulations in controlling the progression of enamel subsurface lesions and to analyze the in vitro fluoride release into artificial saliva.	48 polished enamel specimens from caries-free molars were demineralized in acidic solution for 96 hours to create surface lesions. Then they were divided into six groups: five treated with different fluoride varnishes (MI Varnish, Clinpro White Varnish, Duraphat, Fluor Protector, Enamel Pro) and one control. Surface microhardness was measured before lesioning, after lesioning, and post-treatment. Specimens were stored in artificial saliva at 37ºC, renewed	MI Varnish showed the highest surface microhardness recovery and fluoride release. All fluoride varnishes significantly improved enamel hardness compared to the control, but only MI Varnish was significantly better than Fluor Protector. Duraphat, Enamel Pro, and MI Varnish released the most fluoride, with MI Varnish having the highest release overall. About 60% of fluoride was	All fluoride varnishes increased fluoride levels in artificial saliva, with calcium phosphate-based varnishes enhancing enamel rehardening. The CPP varnish was the most effective in reducing demineralization and releasing fluoride.

		daily. About 10 mg of varnish was applied per sample. Fluoride release was measured at 2, 24, 48 hours, and 7 days with an ion-selective electrode; After 7 days the varnish was removed and samples were incubated 24 hours in fresh saliva for a final fluoride measurement.	released within the first 24 hours. Fluor Protector and Clinpro White had the lowest fluoride release.	
Nahum [75]	Assessment of fluoride release from three varnishes and a resin-modified glass ionomer (RMGI) material.	Three varnishes were used: Fluor Protector (FP), Duraphat (DP), Clinpro White Varnish (CWV) and Giomer (PRG-Barrier Coat) and RMGI (Clinpro XT) were applied to 40 pieces of human tooth in five groups. The samples were then stored for 30 days. Measurements were taken on days 1, 2, 5, 15 and 30. Fluoride release from the varnishes was measured using an ion-selective electrode. After 30 days, the samples were stored in sodium fluoride gel to recharge them. The renewed release was then measured after 24, 48 and 72 hours.	3 varnishes released the most fluoride on day 1. Then there was a decrease except for FP (constantly the lowest amount of released F). On day 1, CWV released the most F. After 24 hours, there was a clear decrease in F release in the varnishes.	The more fluoride a varnish contains, the more it releases into saliva. Regardless of the fluoride carrier, varnish releases the most fluoride for up to 24 hours, after which it becomes a source of small amounts of fluoride.
Asian [76]	Evaluation of fluoride release from 4 varnishes.	Four types of varnish were used: Duraphat, Clinpro White Varnish, Fluor Protector and Varnal Control. These were applied to 44 enamel samples in total. Thirty milligrams of varnish were applied to each sample. Measurements were taken daily in week 1 and then weekly for six	All varnishes released the most fluoride (F) on day 1. Clinpro released the most. Duraphat released the most fluoride (F) stably throughout the entire study. The more viscous the varnish, the more fluoride it releases; the less	The viscosity and wettability of the varnish influence the release of F from the varnish.

		weeks using an ion-selective electrode. Viscosity and wettability were also measured.	viscous it is, the less fluoride it releases.	
Thakur [1]	Estimating how much fluoride different preparations will release into artificial saliva.	A total of 96 human teeth were used and three different preparations were applied: SDF, bifluoride and APF gel. The samples were then placed in artificial saliva. The amount of fluoride released was measured using an ion-selective electrode after 24 hours, seven days and 14 days.	Of all the preparations, the varnish released the most fluoride 24 hours and 14 days after application. After 14 days, the fluoride varnish released the most fluoride.	Varnish seems to be the most effective preparation for the prevention of caries among the three tested due to the highest amount of released fluoride.
Sidhu [22]	The study aimed to assess and compare the fluoride release behavior of four different calcium - phosphate based 5% NaF varnishes complexes over a period of 6 months.	An in vitro study was conducted using 75 extracted non-carious premolars, divided into 5 groups. Four groups coated different fluoride varnishes: MI Varnish™ (with CPP-ACP), Clinpro White Varnish (with f-TCP), Embrace Varnish (with xylitol-coated calcium phosphate), and Enamel Pro Varnish (with ACP). The fifth group served as control. Fluoride release into artificial saliva (50 ml) was measured at 1 day, 1 month, 3 months, and 6 months using the SPADNS spectrophotometric method (570 nm).	Clinpro White Varnish showed the highest total fluoride release across all time points. MI Varnish demonstrated the best long-term release. Embrace Varnish exhibited rapid decline after one month, while the others kept higher release for three months before decreasing.	The study concludes that differences in excipient ingredients markedly affect the fluoride release behavior of varnishes. MI Varnish™ is more suitable for long-term caries protection, while Clinpro White Varnish is better for immediate fluoride delivery.
Attiguppe [2]	The study aimed to assess and compare the demineralization inhibition, fluoride release over time, and antibacterial activity of two fluoride varnishes:	An in vitro study was performed using 24 extracted premolars sectioned into buccal and lingual halves. The buccal halves were tested for demineralization	The MI varnish released significantly more fluoride at all time points (cumulative: 4.19 ppm vs. 3.2 ppm for Fluor Protector) and demonstrated a	Adding CPP–ACP to fluoride varnish enhances its anticariogenic properties by increasing fluoride release, improving enamel

	MI varnish (with CPP–ACP) and Fluor Protector varnish (without CPP–ACP)	resistance using polarized light microscopy after artificial caries challenge, while the lingual halves were tested for fluoride release using a fluoride ion-specific electrode. Antibacterial activity against Streptococcus mutans was evaluated using the disk diffusion method.	stronger inhibition of demineralization (mean lesion depth: 79.78 µm vs. 119.2 µm). It also exhibited greater antibacterial activity (inhibition zone: 24.75 mm vs. 15.25 mm) After 30 minutes: <br>MI varnish: 0.71 ± 0.74 ppm Fluor Protector: 0.36 ± 0.05 ppm Cumulative release after 1 month: <br>MI varnish: 4.19 ± 0.41 ppm Fluor Protector: 3.2 ± 0.19 ppm Statistical significant difference: $p < 0.0001$	remineralization, and boosting antibacterial effects against S. mutans.
Virupaxi [29]	The study aimed to assess and compare the long-term fluoride release from three fluoride varnishes—Clinpro XT, Fluoritop SR, and Fluorprotector—by measuring fluoride concentration in artificial saliva over a period of six months.	An in vitro experiment was conducted using 24 extracted primary anterior teeth, divided into four groups: three test groups and one control. The varnishes were applied to a defined window on the tooth surface. The teeth were then stored in artificial saliva, and fluoride ion levels were measured at 1 week, 1 month, 3 months, and 6 months using a fluoride ion-selective electrode.	All varnishes released fluoride, with the highest release observed in the first week. Clinpro XT showed the most consistent and prolonged fluoride release throughout the six-month period. Fluoritop SR released the most fluoride initially but dropped significantly over time. Fluorprotector had the lowest fluoride release across all time points. The differences among the groups were statistically significant.	Clinpro XT, a resin-modified glass ionomer-based varnish, demonstrated superior and sustained fluoride release compared to the conventional products. These findings suggest that newer varnish formulations may provide enhanced and prolonged caries prevention through continuous low-level fluoride exposure.
Rirattanapong [79]	The objective of this study was to	Twenty-five extracted, primary incisors were	All fluoride varnishes showed	The addition of calcium phosphate

	evaluate and compare the fluoride release over time from different fluoride varnishes, including those enhanced with calcium phosphate, when applied to primary teeth under in vitro conditions.	divided into five groups, including a no-treatment control. Each test group received a different varnish: Duraphat (5% NaF), Clinpro White (5% NaF + TCP), Enamel Pro (5% NaF + ACP), and a freshly prepared Mahidol varnish (5% NaF + TCP). A standardized area on each tooth was exposed and treated with varnish, then immersed in artificial saliva. Fluoride release was measured at multiple time points using an ion-selective electrode.	measurable fluoride release, with the Mahidol varnish exhibiting the highest initial release. Over three months, Duraphat and Clinpro White maintained the highest sustained fluoride levels, while Enamel Pro released less fluoride overall. The control group had no detectable fluoride release. Statistically significant differences were found among the varnish types.	compounds did not consistently enhance long-term fluoride release. Duraphat and Clinpro White demonstrated the most stable and prolonged fluoride release profiles, suggesting their potential for long-term caries prevention in primary teeth.
Ritwik [80]	To analyze the short-term fluoride release patterns from 4 commercially available fluoride varnishes over a 48-hour period.	50 extracted permanent teeth were divided into 5 groups (n=10): four fluoride varnishes (Premier Enamel Pro, Colgate PreviDent, Omni Vanish, Omni Vanish XT - all 5% sodium fluoride) and controls. Teeth were sealed except for 5x5mm windows, weighed, treated per manufacturer instructions, then immersed in 3ml artificial saliva (pH 7.2) at room temperature. Samples were transferred to fresh saliva at 1,2,4,8,12,24,48 hours. Fluoride release measured by ion-selective electrode, expressed as ppm and weight-adjusted.	All varnishes showed declining release rates over 48 h. Premier Enamel Pro (EP) had the highest initial fluoride release and maintained highest levels through 8 h. 3 varnishes (EP, Colgate PreviDent, Omni Vanish) reached fluoride release plateau at 4 h, while Omni Vanish XT showed sustained release without plateau throughout the study period. Omni Vanish XT demonstrated lowest initial release (first 4 h) but higher sustained release after 4 h compared to other products.	3 products (Premier Enamel Pro, Colgate PreviDent, Omni Vanish) plateaued at 4 h, indicating safe tooth brushing resumption. Omni Vanish XT demonstrated sustained release without plateau. Premier Enamel Pro had highest initial fluoride output. Results provide evidence-based timing for post-application oral hygiene recommendations.
Castillo [30]	To compare fluoride release from two application	Primary molar enamel slabs (5x5mm) randomly assigned to	Triple application released more total fluoride than single	Triple fluoride varnish application in one week provides higher

	protocols: single application versus three applications in one week period.	single application (n=5) or triple application within one week (n=5), plus controls (n=4). Duraphat varnish (5% sodium fluoride, 30mg/specimen) applied at baseline and days 2,4 for triple group. Specimens immersed in buffered calcium phosphate solution (pH 6) on shaker table. Fluoride release measured weekly for 21 weeks using fluoride electrode with TISAB buffer.	application (34.9 vs 23.7 μmol). Peak release occurred at week 1 for both groups (9.56 vs 5.73 μmol). Triple application showed slower release rate (-0.026 vs -0.058 slope) and maintained higher release from weeks 8-21. After 21 weeks, triple application retained 68.1% vs 35.1% for single application. Controls released <1 μmol.	total fluoride release, slower release rates, and longer fluoride availability compared to single application.
Castillo [81]	To assess fluoride release patterns between two commercially available fluoride varnishes (Duraphat and Duraflor) over six months.	Primary molar enamel slabs (5x5mm) assigned to Duraphat (n=9), Duraflor (n=9), or controls (n=5). Both varnishes (5% sodium fluoride, 30mg/specimen) applied. Samples immersed in buffered calcium phosphate solution (pH 6.0) with weekly transfers for 6 months. Fluoride release measured using calibrated electrode with TISAB buffer - daily in week 1, then weekly.	No significant difference in total fluoride release between products at study end (Duraphat: 25.1±4.9 μmol vs Duraflor: 20.2±14.7 μmol). Duraphat released 67% and Duraflor released 56% of applied fluoride. Duraphat released more fluoride than Duraflor from week 4. Duraphat showed a slower release rate (slope -0.06±0.01) compared to Duraflor (-0.09±0.02). Duraflor showed greater variability in release (range: 3.1-46.8 μmol) than Duraphat (range: 18.6-33.9 μmol). Duraphat continued to release until week 28, while Duraflor stopped at week 19.	Both varnishes released fluoride for 5-6 months with comparable total output, but with had different pattern. Duraphat provided a more consistent, prolonged release with less variability, while Duraflor showed higher initial release but greater product inconsistency.

Table S2. Quality assessment of included studies.

Authors	1. Is It Clear in the Study What Is the 'Cause' and What Is the 'Effect'?	2. Were the Participants Included in Any Comparisons Similar?	3. Were the Participants Included in Any Comparisons Receiving Similar Treatment/Care, Other than the Exposure or Intervention of Interest?	4. Was There a Control Group?	5. Were There Multiple Measurements of the Outcome Both Pre and Post the Intervention/Exposure?	6. Was Follow up Complete and If Not, Were Differences Between Groups in Terms of Their Follow up Adequately Described and Analyzed?	7. Were the Outcomes of Participants Included in Any Comparisons Measured in the Same Way?	8. Were Outcomes Measured in a Reliable Way?	9. Was Appropriate Statistical Analysis Used?
Barrera-Ortega [3]	Yes	Yes	Yes	Yes	Yes	Yes	Yes	Yes	Yes
Singh [74]	Yes	Yes	Yes	Yes	Yes	Yes	Yes	Yes	Yes
Okuyama [77]	Yes	Yes	Yes	Yes	No	Yes	Yes	Yes	Yes
Pańczyszyn [78]	Yes	Yes	Yes	No	Yes	Yes	Yes	Yes	Yes
Yildiz [4]	Yes	Yes	No	Yes	Yes	Yes	Yes	Yes	Yes
Nahum [75]	Yes	Yes	No	No	Yes	Yes	Yes	Yes	Yes
Asian [76]	Yes	Yes	Yes	Yes	Yes	Yes	Yes	Yes	Yes
Thakur [1]	Yes	Yes	Yes	Yes	No	Yes	Yes	Yes	Yes
Sidhu [22]	Yes	Yes	Yes	Yes	Yes	Yes	Yes	Yes	Yes
Attiguppe [2]	Yes	Yes	Yes	No	Yes	Yes	Yes	Yes	No
Virupaxi [29]	Yes	Yes	Yes	Yes	No	Yes	Yes	Yes	Yes
Rirattanapong [79]	Yes	Yes	Yes	Yes	No	Yes	Yes	Yes	Yes
Ritwik [80]	Yes	Yes	Yes	Yes	Yes	Yes	Yes	Yes	No
Castillo [30]	Yes	Yes	Yes	Yes	No	Yes	Yes	Yes	No
Castillo [81]	Yes	Yes	Yes	Yes	No	Yes	Yes	Yes	No
